# Influence of the Interlayer Temperature on Structure and Properties of Wire and Arc Additive Manufactured Duplex Stainless Steel Product

**DOI:** 10.3390/ma13245795

**Published:** 2020-12-18

**Authors:** Nikola Knezović, Ivica Garašić, Ivan Jurić

**Affiliations:** 1 Faculty of Mechanical Engineering, Computing and Electrical Engineering, University of Mostar, Matice hrvatske bb, 88000 Mostar, Bosnia and Herzegovina; 2 Faculty of Mechanical Engineering and Naval Architecture, University of Zagreb, Ul. Ivana Lučića 5, 10000 Zagreb, Croatia; ivica.garasic@fsb.hr (I.G.); ivan.juric@fsb.hr (I.J.)

**Keywords:** wire and arc additive manufacturing (WAAM), duplex stainless steel (DSS), interlayer temperature, ferrite amount, porosity, hardness

## Abstract

WAAM (wire and arc additive manufacturing) is becoming an increasingly popular method to produce components from metals, which are usually not so suitable for conventional production methods. One of the good examples is duplex stainless steels (DSSs), which are quite complex for welding and machining. Excessive ferrite amount is a common problem for them and controlling an interlayer temperature could offer a solution. However, using too low interlayer temperature will slow down the whole process and compromise one of the WAAM’s main advantages—the high productivity. The aim of this study is to find the relationship between interlayer temperature and process duration and to determine the influence of the interlayer temperature on product structure and other properties. Three samples (walls) were made using different interlayer temperatures (50 °C, 100 °C and 150 °C) and they were tested to analyze their surface texture, chemical composition, ferrite amount, the appearance of porosity and the hardness. Ferrite amount was higher and there was more porosity on lower interlayer temperatures, while there is no significant difference between surface texture and chemical composition for the samples. Considering the fact that higher interlayer temperatures provide a faster process, they should be preferred to produce duplex stainless steel products.

## 1. Introduction

Growth and evolution of the modern industries always bring the need for developing and researching the new technologies and the novel production methods. In the latest years, AM (additive manufacturing) technologies caught the eye of researchers, due to their possibility to produce the complex geometries with more design freedom, high productivity and less waste. However, the majority of these technologies are still limited to produce only the prototypes and they often use only polymer materials. The appearance of the wire and arc additive manufacturing (WAAM) technology and its development could be a potential solution for some problems and challenges, which are the main drawbacks for conventional AM technologies, like excessive porosity, limited choice of the materials, low mechanical properties, etc. [1,2,3,4].

WAAM has been investigated more detailed since the 1990s, although first related patent dates almost 100 years ago, from 1925 [5]. Since it uses welding wire as additional material and electric arc as a heat source, it is considered as a combination of welding and additive manufacturing. It means that the general WAAM system could be constructed combining common welding equipment (power source, shielding gas, welding torch, wire feeder, etc.) and some equipment that can provide movement (robotic hand or CNC machine) [6].

The thing that distinguishes WAAM compared to conventional AM technologies is the fact it can produce fully functional metal products with welding wire only, which means it is not necessary to buy and store large amounts of raw material. For example, according to source [7], projected demand for the aerospace industry is 20 million tons of billet material in the next 20 years if conventional production methods are to be used. Using WAAM could reduce the need for billet material greatly. Considering that, it is understandable that most of the research related to WAAM try to include particular materials, which are used in the aerospace industry (titanium, stainless steels, nickel-based alloys, etc.) [1,8,9,10]. Some other advantages that make this method more and more popular are the high deposition rates (range from 50 g/min to 130 g/min), lower cost compared to some other AM technologies, safer work, and almost unlimited part size (basically, the only limit is the size of the working area of the robotic hand or CNC machine used to provide motion) [11,12].

However, some issues and problems are still to be resolved and they are mostly welding-related. Different papers [5,13,14,15] recorded problems with anisotropic mechanical properties, distortions and residual stresses induced with excessive heat input, and relatively poor surface finish (additional machining is mostly necessary). Solutions for reducing these problems exist, but further research is still needed.

Duplex stainless steels are the part of the stainless steels family, the class of the steels known for their corrosion resistance, which they were named after. Due to their mixed austenitic/ferritic microstructure (roughly, they consist of 50% of both phases) they are amongst the most interesting stainless steels for research purposes [16,17,18]. They have combined lattice arrangement, where austenite provides good toughness and pitting corrosion resistance, while ferrite ensures good tensile strength and resistance to general corrosion [19]. Their microstructure is formed by primary crystallization of ferrite and its partial transformation austenite during the cooling process [20]. Since their microstructure is very susceptible and prone to changes when exposed to heat input and repeated cycles of heating and cooling, welding of DSS is always a demanding process and their application is sometimes limited, especially in the as-welded condition [18,21,22].

Those kinds of problems were the main drawback for the first generation of DSS, which was produced in 1930, but it could not be used in the welded condition since excessive ferrite amount in the weld metal and HAZ (heat affected zone) has deteriorated toughness and corrosion resistance [20]. Some solutions for that problem came out later, and today’s DSS are usually classified into five groups, divided by their chemical composition and corrosion resistance (the alloy content determines their corrosion resistance). The least alloyed are called lean duplex grades and the highly alloyed are hyper duplex grades [16]. Their major alloying elements are divided into two groups: alphagenous elements (Cr, Mo, Si, Ti, etc.), which promote the formation of ferrite and gamagenous elements (Ni, Mn, C, N, Cu, etc.), which promote the formation of austenite. Most important alloying elements are Cr and Ni, which are also present in the largest amount [17].

A lot of mechanical and corrosion properties could be good enough when microstructure is obtained where austenite and ferrite both are ranging from 30% to 70%, including welded metal. However, roughly equal amounts are usually required, where slightly more austenite is preferred, due to better toughness. To obtain the desired microstructure, it is essential to control the cooling rate during welding (if it is too fast, more ferrite will be formed). Composition of shielding gas and the chemical composition of the substrate and additional material are also important factors to reach that goal [23].

Limitation for application of the DSS is the problems and the difficulties that occur during welding and machining. Due to repeated cycles of heating and cooling, the formation of harmful intermetallic phases is sometimes inevitable, particularly in highly alloyed grades. Phases like sigma (σ), chi (χ), alpha prime (α’) and chromium nitride (Cr_2_N) have been reported elsewhere and their detrimental effect is well-known [24,25]. Besides that, they are generally difficult to machine, due to their low sulfur content and high yield strength. Every material in engineering is usually evaluated by the possibility to manufacture products using it with minimum cost and problems during fabrication, which is a drawback for the DSS, because of the welding and machining issues [20]. However, WAAM could offer a solution as a novel and an innovative technology, since it is able to produce almost fully functional and near-net-shape products where machining is necessary only as additional post-process. WAAM also could reduce welding-related issues, because welding wire is used solely (there is no need to look for matching additional material). Other issues have to be investigated further in order to be reduced, but some solutions to solve anisotropy [26] or distortion [7] problems are already found. Additionally, the use of WAAM for production of DSS parts is becoming popular lately [27,28,29] which indicates a need to improve findings in this area.

Fast production is one of the main advantages of the WAAM and it can be partially controlled through adjustment of welding speed [30,31,32,33,34]. However, increasing welding speed too much causes process instability and poor part quality, thus it has to be held in an optimal range [30]. Another way to speed up the process is to reduce the time between two adjacent passes, which means higher interlayer temperatures are necessary. A negative consequence of using higher interlayer temperatures in welding is shortening the time necessary for the transformation of ferrite to austenite, which can lead to excessive ferrite amount. For example, the study [35] showed lower amounts of N_2_ and lower impact toughness using lower interpass temperatures with DSS, which could be a consequence of higher ferrite account. However, it is well-known [27,29,36,37] that DSS parts could be produced with a lower ferrite amount, which can even fall under 30%. Since it was already proven that WAAM could produce DSS parts with acceptable microstructure and lower ferrite amount (despite repeated cycles of heating and cooling), the aim of this study is to find the highest possible interlayer temperature, which will still have the parts produced with acceptable lower ferrite amount. Higher interlayer temperature, consequently, means the idle times are shorter and the whole process is faster.

One of the drawbacks of the WAAM process is its susceptibility to porosity, which is well reported in the literature [5,6,8,38,39,40,41]. The porosity does not occur as a rule, but these papers mention poor wire handling, substrate contamination (moisture, dust, etc.), inappropriate parameters like insufficient gas flow or high travel speed or absorption of different gases—mostly hydrogen and nitrogen—in molten weld pool due to poor shielding gas protection, as the reasons why porosity can be present. This latest reason could be the most important, since it is process-related, while others are mostly human errors. However, to the authors’ best knowledge, there is no paper that could prove any of these statements. Since our first try-outs with WAAM technology have shown significant porosity at the lateral external sides, our other goal is to find out is there any porosity at the inside of the part and whether it is its amount compliant with requirements of the appropriate standards.

Surface roughness is analyzed in order to see if there are any significant differences caused by the different interlayer temperatures.

Chemical composition of the product is analyzed to check are there any changes in nominal composition given by the wire manufacturer.

Hardness testing was done to see if it is possible to produce the WAAM duplex stainless steel part, which will fulfill the requirements of appropriate standards for hardness.

## 2. Materials and Methods

**Figure 1:**
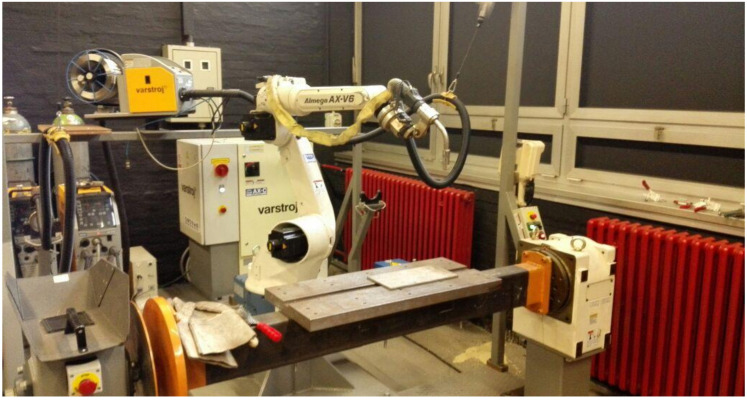
Wire and arc additive manufacturing (WAAM) system used for this study.

An experiment was carried out in Welding Laboratory at the Faculty of Mechanical Engineering and Naval Architecture, University of Zagreb. WAAM system was constructed particularly for this study, by combining welding robotic hand OTC Almega AX-V6 (six axes of freedom) with MIG welding machine DP-400 (conventional power source) and wire feeder CM-7401. All three devices were supplied by Daihen Varstroj (Lendava, Slovenia). Welding wire used as a feedstock was 1.2 mm diameter duplex stainless steel solid wire Avesta 2205 (Voestalpine Böhler Welding—Hamm, Germany) which is designed for welding DSS grade 2205 or 1.4462). Shielding gas mixture was prepared by Messer Croatia Plin (Zaprešić, Croatia), containing 98% Ar and 2% N_2_. The substrate was austenitic (grade 304 or 1.4301) stainless steel plate, with dimensions 300 mm × 150 mm × 8 mm. This WAAM system is shown in Figure 1.

It was essential to find a set of appropriate welding parameters, which will provide the production of samples with a stable and smooth process. Additionally, the important requirement to fulfill was arc energy (*AE*). According to source [20] and manufacturer’s recommendation, arc energy for this DSS grade should be at least 0.4 kJ/mm, calculated using Equation (1):

(1) $$AE=\frac{I\cdot V}{1000\cdot v}[\frac{kJ}{mm}]$$

where *I* is the welding current, *V* is the voltage and *v* is the welding speed. This equation was used mainly due to experimental limitation—the only available equipment at the moment of this study was the one that could measure the values necessary for this equation. The more accurate and more used method lately is the method with instantaneous power, mentioned in the papers [42,43,44]. However, since *AE* was used only to see if the requirements for this DSS grade are met and not for any other reasons, Equation (1) is still satisfying for this purpose.

Different combinations of parameters were tried, but only four sets fulfilled the requirements. The chosen set was the one amongst them who provided the most stable process and gave the smoothest bead. These sets are shown in Table 1.

One can see there are some differences in welding speeds between the first two layers and the others. Changing the welding speed was necessary since some humps and instability of the electric arc appeared immediately at the beginning of the second layer deposition. Humps can be explained by the motion caused by surface tension pinching force [45]. Another reason could be slower heat dissipation than it was during the first layer deposition when the substrate plate absorbed a greater amount of heat. These problems were even worse for every next layer, but increasing the welding speed was a satisfying solution to reduce that. Increasing the welding speed for higher layers has another positive outcome, as reported in [30,31,32,33,34]—decreasing the wall width and reducing overflow of the molten metal on both sides of the wall. However, the increase of the welding speed leads to lower arc energy, so 27 cm/min had to be a limiting value in order to keep the arc energy above 0.4 kJ/mm. Interlayer temperatures were chosen according to sources [14,15] and the manufacturer’s recommendations for this DSS grade (maximum interpass temperature is limited to 150 °C).

With these values chosen, samples (walls) could be made, using a standard WAAM layer-by-layer manner. The robotic hand used generated tool path to deposit the first layer on the substrate plate. Then the torch was moved up for suitable height—approximately 2 mm—and the second layer was deposited over the first one, the third one over the second one, etc. After every layer, the deposition direction was reversed to reduce the distortion of the finished part as much as it is possible. Every layer is left to cool on the temperature chosen for the particular part before deposition of the next layer. The wall made with the lowest interlayer temperature (50 °C) was W1, the wall made with medium interlayer temperature (100 °C) was W2 and the wall made with the highest interlayer temperature (150 °C) was W3. The interlayer temperature was measured using infrared thermometer Fluke 568 (Fluke Corporation—Everett, WA, USA). Since it is a contactless device, it was easy to manage to measure at the whole length of the layer. On the other side, it was almost impossible to obtain the temperature equilibrium through the whole length of the layer, so these temperature values were not always fixed (for example, when the majority of the layer on W1 reached desired 50 °C, some parts of the wall were on 48 °C or 52 °C). However, these differences were minor for all layers and all the walls, so the fixed values were kept for easier tracking. Room temperature was constant and the same for all walls. Idle times between the layers were different for all three walls, as was expected since they were dependent on interlayer temperatures.

Finished walls were then sent for 3-D scanning with DAVID SLS-3 device (David Vision Systems - Koblenz, Germany) in order to get the appropriate file formats for testing surface roughness using software Mountains Map Premium 7.4 (Digital Surf). Results were evaluated according to standard ISO 25178-2:2012—geometric product specifications (GPS)—surface texture: areal [46] and source [47], which gives guidelines for assessment and application of results obtained. Shortly, this analyzing method is more advanced than the classical 2-D method because the measured area is actually three-dimensional, which means this measurement is more comprehensive. It is also more precise statistically since it takes more parameters into consideration.

After that, specimens for microstructure observations and ferrite amount testing were extracted and then the walls were milled from the lateral sides to remove the material excess. 

After additional milling, samples were tested with the radiography method (RTG) to detect any porosity. The system used for testing consists of RTG device Balteau GM 300D (Balteau NDT SA—Oupeye, Belgium), scanner VMI 5100MS-C (VMI NDT—Pensacola, Florida, USA) and imaging plate Carestream Industrex Flex Blue (Carestream NDT—Rochester, New York, USA). Radiograms were evaluated using wire image quality indicator 13FEEN (NDT Consultants—Coventry, UK). 

Chemical composition of walls was tested with the fluorescence spectroscopy method, using Vanta^TM^ M handheld XRF analyzer (Olympus Europa SE & Co. KG—Hamburg, Germany). The sample was taken for each wall and tested on three points. The points were then averaged in order to obtain the results. Finally, samples for hardness testing were taken from each wall. They were ground after cutting with SiC paper (granulations P120, P320, P500, P1000, P2000 and P4000, respectively) and etched in 10% oxalic acid for three minutes.

Specimens for ferrite amount analysis were polished and etched with the ethane dicarboxylic acid (C_2_H_2_O_4_), using 10 V voltage and in the duration of 20 s. Images were taken with the Olympus GX51 (Olympus Europa SE & Co. KG—Hamburg, Germany) inverted metallurgical microscope, with the magnification of 200×. Images were analyzed using software ImageJ (version 1.52r). Lately, point counting analysis using ferritscopes, which measure ferrite number is more present, but some newest articles suggest image analysis as a more precise and more reliable method [48,49]. This method’s main drawback is its dependency on etching quality. If austenite and ferrite are not distinguished good enough, the software could not tell the difference precisely and there is a possibility for the mistakes. Usually, there is an option to set the threshold manually, but then the results will depend on the operator’s judgment. To compensate for a possible mistake and to get the most accurate results, 12 images were taken for each sample on different places from the bottom to the top of the sample. For a particular sample, the ferrite amount was calculated for each image and the results were averaged to get the value for that wall.

**Figure 2:**
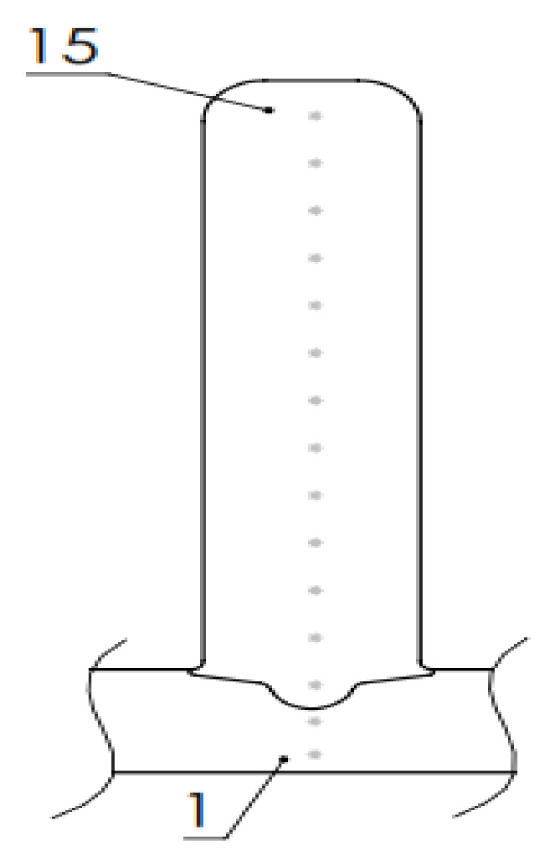
Points for hardness measuring.

Hardness testing was done using Vickers method and Brivisor KL2 (Reicherter). Figure 2 shows the pattern of 15 points where hardness was measured.

Three different measurements were executed at each point and then the values were averaged and recorded. Force of 98.04 N (10 kp) was used to obtain HV10 hardness results. Thirteen results—without the first 2 points, which belong to the substrate—were then averaged for each wall to get the average hardness value. Additionally, the hardness profile was made for every wall, which shows the distribution of hardness values through the part.

## 3. Results and Discussion

### 3.1. General Observations

**Figure 3:**
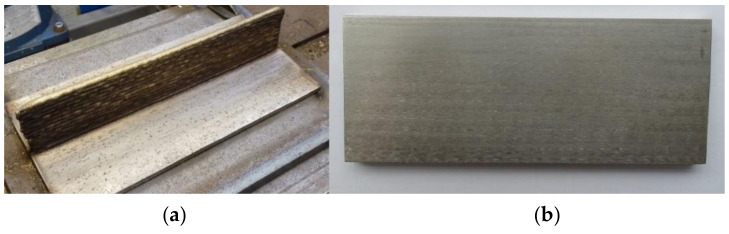
Finished WAAM wall (**a**) and the same wall after additional milling (**b**).

Figure 3 shows the finished wall (a) and the same wall after additional milling (b).

The whole manufacturing process was quite stable, which is the first important finding. This set of parameters can be used to successfully produce DSS parts without significant problems like major distortions. However, additional testing of mechanical properties is suggested to see if they are satisfying and if there is any anisotropy. 

All walls had satisfying geometrical characteristics since they stayed perpendicular to the substrate plane. There was no bending to the left or right, which means the robotic hand is a convenient solution for manufacturing similar parts. Some minor distortion occurred along the vertical axis, where the accumulation of the layers “pulled” the ends of the substrate up and bent it despite rigid clamping, causing the wall to be higher 1–2 mm on the ends than in the middle. The first and last few layers were planned to be cut anyway and this should not be considered as a problem. However, residual stresses were not assessed in this research and they should be one of the things to focus on in further research.

**Figure 4:**
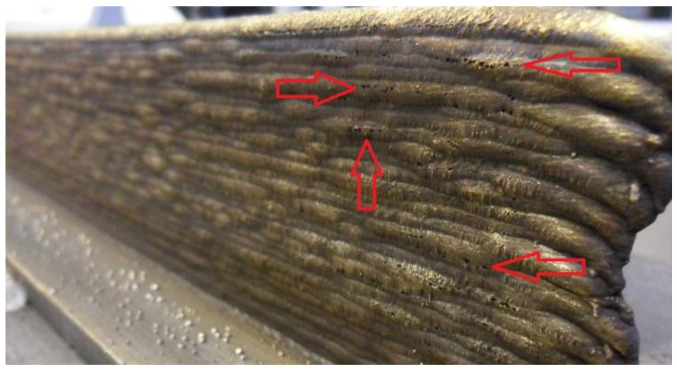
Porosity in one of the walls.

The greatest issue, which is seen without any additional testing is porosity, shown in Figure 4. Groups of pores are located at the external sides. Pores are more present at higher layers, probably because the protection of the shielding gas gets weaker due to its loss in the surrounding air. For the lower layers shielding gas is deflected from the substrate, thus creating more complete protective surrounding. Additionally, a possible explanation could be the turbulences, which can occur due to the hot air. It is easier than the cold air and it ascends along the wall, “breaking” the shielding gas envelope. Even the increased welding speed could be the reason since it could deteriorate shielding gas protection [30]. These are only assumptions, but they could be interesting and worth enough to be researched further. For example, a kind of shielding gas chamber could be designed and introduced to the process. This idea could help to stop—or at least reduce—shielding gas loss in surrounding air, thus improving the protection of the wall and prevent excessive forming of the pores. However, this issue supports the idea to test milled walls to check for possible porosity at the inside. The porosity should be reduced and there is a room for process improvement during the future research, and the guideline could be the idea presented in papers [50,51], where the appropriate shielding gas device was designed to improve the protection of the process.

Prior to any testing, the most important information was manufacturing time for different interlayer temperatures, which is shown in Table 2.

### 3.2. 3-D Analysis of the Surface Texture

**Figure 5:**
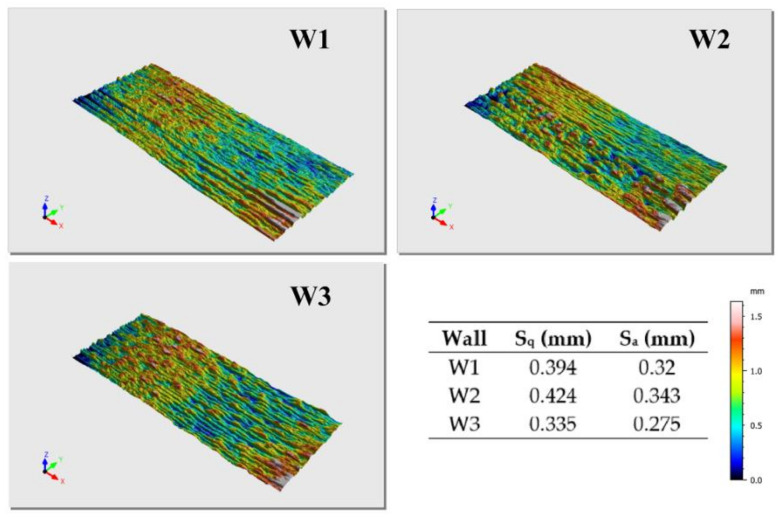
Results of the 3-D analysis of the surface texture.

Three-dimensional analysis of the surface texture was done on one side of every wall due to time savings since it is assumed that both sides of the same wall are almost equal in the terms of surface roughness. Figure 5 shows 3-D scanned walls and parameters obtained after the 3-D scans were processed. The most important factors to use in the evaluation of the walls are S_q_ (root mean square height of the surface) and S_a_ (arithmetical mean height of the surface). If their value for the certain wall is significantly higher than for the others, it means the surface is rougher and more difficult additional machining is to be expected (friction coefficient tends to increase) [52]. According to the results, W3 has the lowest surface roughness and it should be slightly easier to machine. However, differences in the results are minor and that was confirmed during machining, where all three walls showed similar behavior. Considering these results, the interlayer temperature does not have any influence on surface roughness.

### 3.3. Chemical Composition

**Figure 6:**
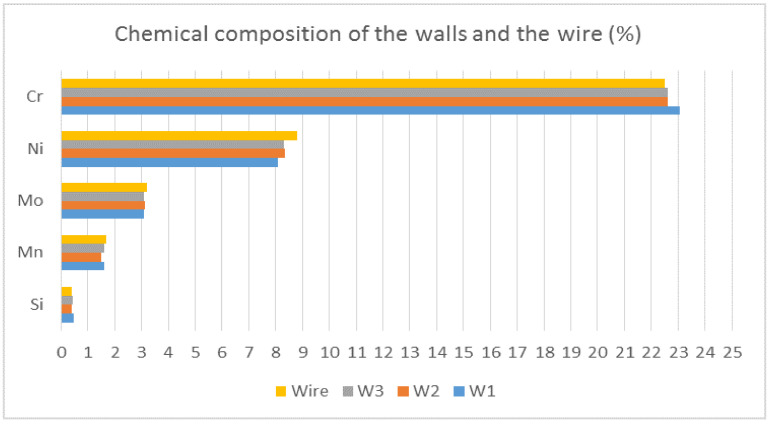
Chemical composition of the walls and the wire.

Results of chemical composition testing are shown in Figure 6. It can be seen there were no significant differences between the three walls. Additionally, none of the walls differed from the wire a lot. Clearly, the interlayer temperature did not affect the chemical composition of the walls and—what is even more important—chemical composition of the wire was not altered significantly by the process, so additional material for future research could be chosen without fear of affecting its chemical composition significantly. However, it is crucial to mention the limitation of the device used—it was not possible to detect the N_2_, which can be higher due to a composition of the shielding gas. Ferrite amount could be a guideline (if it is lower, N_2_ could be higher if there is more austenite), but further research must focus on finding a way to measure the N_2_.

### 3.4. Ferrite Amount Analysis

Table 3 shows the ferrite amount for each wall, along with the standard deviation.

**Figure 7:**
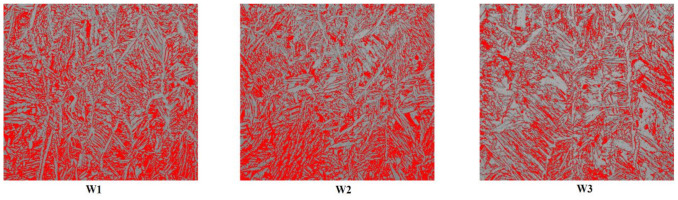
Example of ferrite amount testing using image analysis.

Clearly, an increase in the interlayer temperature caused the ferrite amount to decrease. It is important to mention that there were no significant differences between ferrite amounts at different layers (heights), which means microstructure was quite homogenous. Figure 7 shows some of the images used for analysis. It is easy to see there was more austenite (grey-colored), which formed longer grains growing in the vertical direction, while ferrite grains (red-colored) were mostly staggered on the sides. Some of the black dots were probably carbides or defects made during the preparation of the samples. However, they could be rarely seen. While wire datasheet claimed there was 45–55% of ferrite, W1 had 38.5% and another two walls had even less. This is accordant with results from [53], where the lower ferrite amount was also recorded at higher interlayer temperatures. The possible reason could be the shorter idle time—while the previous layer was not cold still, the subsequent layer was already heating it, giving enough time for austenite to form. At lower interlayer temperatures transformation to ferrite could occur while the layer was cooling and the heat input during the next layer deposition was not high enough to cause the transformation to austenite, thus leaving the part with higher ferrite amount. Both phases should be present in roughly equal amounts in DSS, but if they are not it is preferable to have more austenite since it tends to increase some important properties (impact toughness or pitting corrosion resistance). If there are requirements for minimum ferrite amount, an approach suggested in [27] could be used. Otherwise, the higher interlayer temperature should be preferable to use since it means it is possible to produce parts faster and they will still have acceptable amounts of ferrite. Lastly, these results are comparable with studies [27,29,36,37], which were mentioned in the introduction.

### 3.5. Radiography Testing

**Figure 8:**
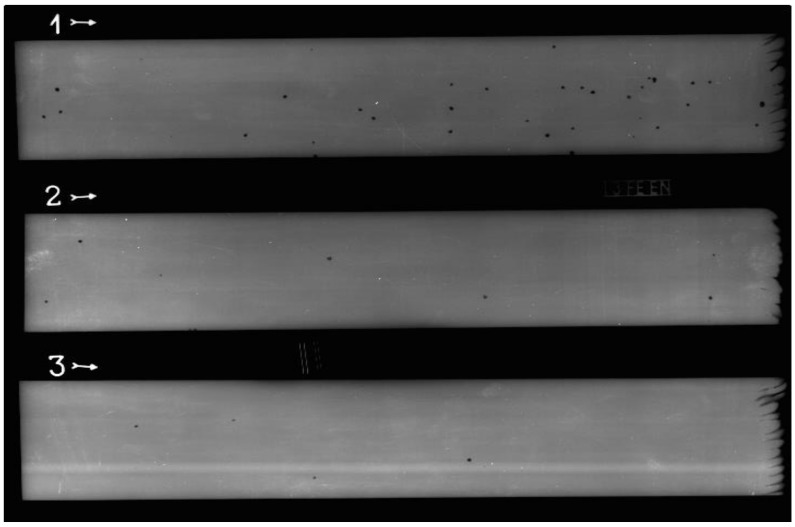
Radiograms of the walls.

Figure 8 shows radiograms obtained with the radiography testing. The smallest pore that could be seen with sensibility used was 0.05 mm in diameter. Since there were still no specific standards for WAAM products, existing standards for weldments were used to classify defects found and to evaluate if they could be acceptable:

- ISO 6520-1:2007—Welding and allied processes—classification of geometric imperfections in metallic materials—Part 1: fusion welding [54];
- ISO 5817:2014—Welding—fusion-welded joints in steel, nickel, titanium and their alloys (beam welding excluded)—quality levels for imperfections [55].

All imperfections found were pores (gas inclusions). Table 4 shows the number of pores for each wall. It is clearly seen that the number of pores increased as the interlayer temperature was lower. The largest pores were close to 1 mm in diameter, which is important for the evaluation of the product.

While porosity at the external sides is not so dangerous—external sides are milled anyway—pores at the inside could cause deterioration of mechanical properties (toughness). According to ISO 6520-1:2007 [54], all these pores could be classified as subgroup 2012—uniformly distributed porosity. To evaluate the acceptability of these imperfections, ISO 5817:2014 could be used [55]. Since there are no specific standards for WAAM products, requirements for multipass welding are the closest to compare with. Acceptance level for the strictest class (B) is a maximum of 2% of weld area covered with porosity. Since the dimension of the wall after machining was 280 mm × 60 mm, its area was 16,800 mm^2^. The greatest pore was 1 mm in diameter, which gave an area of 0.785 mm^2^, roughly (pores were round mostly). In the worst case—for W1—porosity covered approximately 0.15% of the wall area, which was far below acceptable values. However, additional testing of mechanical properties—especially impact toughness—should be done to check if there are some issues that could be caused by porosity. The possible reason for fewer pores at the higher interlayer temperature could be related to the ferrite amount. The solubility of the nitrogen in ferrite is slightly lower at the lower temperatures—according to [56]—so it is possible that some nitrogen from the shielding gas has been trapped in the wall with higher ferrite amount (since the walls made with lower interlayer temperatures have more ferrite and more pores). Additionally, higher interlayer temperatures provide less time for gas to completely form the inclusions in solidified metal, leaving the walls with less porosity. According to all of the findings related to porosity, higher interlayer temperatures should be favored in this process. In accordance with the studies [5,6,8,38,39,40,41] mentioned, porosity was not totally avoided, but it was at least kept in the acceptable ranges inside the part.

### 3.6. Hardness Testing

**Figure 9:**
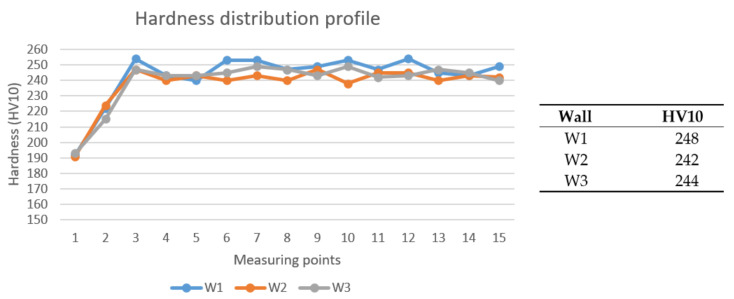
Hardness distribution profiles.

Hardness distribution profiles and average hardness values are shown in Figure 9. One can see there were no significant differences between the parts. Only W1 hardness was slightly higher, but it was a minor difference. Additionally, distribution profiles did not have greater deviations, which means hardness was uniformly distributed across the parts’ section (similar to ferrite amount, it means the parts were quite homogenous across their sections). However, no pattern can be found to say the interlayer temperature could affect the hardness of the parts.

Contrary to the results presented in the studies [57,58,59]—where higher hardness exhibited at lower porosity levels—W1 sample had the highest hardness and highest percentage of porosity. This could seem unusual, but it is important to point out that the hardness values differed only slightly (6 HV10) and porosity percentage differed even less (less than 0.15%). At the studies mentioned, hardness values varied even more than 50 HV10, and porosity percentage almost up to 10%. Additionally, W2 and W3 hardness values did not follow that pattern. Therefore, this anomaly could be due to the measurement process—it is possible that—during measuring—tool tip tapped some nitrides or carbides (which could be present, but they were not subject of this study). Additional researches have to be done in order to prove this.

Standards EN 10088-3:2014 [60] and EN 10088-5:2009 [61], which define technical delivery conditions for different types of DSS products, require a maximum of 270 HB for products thinner than 160 mm. It is roughly equal to 277 HV, which means only the last few layers did not fulfil that requirement. However, since the first few and last few layers should be cut during machining (prior to the application of the part), it is important to know that main part of the wall have satisfying and homogenous properties.

## 4. Conclusions

As a result of this study, conclusions were as follows:

1. These parameters are appropriate to build the wall with satisfying geometrical characteristics and without major distortions, but porosity could not be avoided. Even if it is true that the majority of the external sides are additionally post-processed and machined, some of the porosity still could be inside. Further research is necessary in order to improve the process. Additionally, since the distortions were minor, the focus for the further research should be on the measuring of the residual stresses.
2. Interlayer temperature does not affect surface roughness at all. Minor differences amongst samples could not be attributed to temperature since there is no regular pattern. All the walls have shown similar behavior during machining, which adds to that.
3. Chemical composition of the walls was similar amongst all of them, but also similar to the composition of the wire. However, there was a limitation of the impossibility of N_2_ detection, and further research should aim to improve that.
4. Ferrite amount analysis has shown there is more ferrite in the walls produced with the lower interlayer temperature. It is important finding directly related with our first objective since now it is proved that it is possible to produce the parts with lower ferrite amount by using higher interlayer temperatures, which consequently means shorter idle time and a faster process.
5. Radiography testing showed there was some porosity inside the walls. Since there are still no standards, which would be suitable for WAAM products, standards used for assessment were the ones that are the closest by nature of the process—standards for weldments. According to them, all the walls were acceptable even using the acceptance levels for strictest class (B) and it was an important finding related to our second objective. However, solutions that may be found to reduce the external porosity could also be helpful to reduce the internal porosity.
6. Hardness values amongst the walls were close and did not have significant differences. An important finding is a fact that these products fulfill the hardness values requirements defined by appropriate standards for duplex stainless steel products. It means we reached our goal and proved it was possible to produce the WAAM parts, which were comparable (in terms of hardness properties) with the products made using conventional methods.
7. Considering production time, ferrite amount and porosity, it is strongly suggested to use higher interlayer temperatures to produce parts using this duplex stainless steel grade. Wall produced using the interlayer temperature of 150 °C was made for more than twice less time than the one with 50 °C while having about 5% lower ferrite amount and significantly less porosity.

However, to better understand the relationship between welding parameters, production process and overall quality of WAAM produced duplex stainless steel part, suggestions for further research include:

- Additional mechanical properties testing (especially impact toughness testing);
- Corrosion resistance testing;
- Measuring of residual stresses;
- Process improvement (for example, designing a chamber to create a better protective environment of shielding gas);
- The possible introduction of on-line non-destructive testing to find potential defects as soon as they appear;
- Altering the cooling rate to see how it could be used to manage the microstructure.

## Figures and Tables

**Table 1:** Chosen set of parameters for samples production.

Welding Parameters	Values
Welding current, *I*	132–136 A
Voltage, *V*	17 V
Wire-feed speed	386 cm/min
Welding speed (1st layer)	25 cm/min
Welding speed (2nd layer)	26 cm/min
Welding speed (3rd–30th layer)	27 cm/min
Shielding gas	98% Ar + 2% N2
Shielding gas flow	17 L/min
Additional material (wire)	Avesta 2205
Wire diameter, Ø	1.2 mm
Contact tip-to-work distance, l	10 mm
Travel angle	90°
Metal transfer mode	Short-circuit

**Table 2:** Manufacturing times for different interlayer temperatures.

Wall	Manufacturing Time
W1	132 min
W2	69 min
W3	48 min

**Table 3:** Ferrite amounts for the walls.

Wall	Ferrite Amount (%)	Standard Deviation (σ)
W1	38.5	0.67
W2	35.8	0.46
W3	33.9	0.70

**Table 4:** The number of pores per wall.

Wall	Number of Pores
W1	33
W2	8
W3	4

## References

[1] DebRoy T., Wei H.L., Zuback J.S., Mukherjee T., Elmer J.W., Milewski J.O., Beese A.M., Wilson-Heid A., De A., Zhang W. (2018). Additive Manufacturing of Metallic Components—Process, Structure and Properties. Prog. Mater. Sci..

[2] Busachi A., Erkoyuncu J.A., Colegrove P., Martina F., Ding J. (2015). Designing a WAAM Based Manufacturing System for Defence Applications. Procedia CIRP.

[3] Mohamed O.A., Masood S.H., Bhowmik J.L. (2016). Investigation of dynamic elastic deformation of parts processed by fused deposition modeling additive manufacturing. Adv. Prod. Eng. Manag..

[4] Vesenjak M., Gačnik F., Krstulović-Opara L., Ren Z. (2015). Mechanical Properties of Advanced Pore Morphology Foam Elements. Mech. Adv. Mater. Struct..

[5] Williams S.W., Martina F., Addison A.C., Ding J., Pardal G., Colegrove P.A. (2015). Wire + Arc Additive Manufacturing. Mater. Sci. Technol..

[6] Busachi A., Erkoyuncu J., Colegrove P., Martina F., Watts C., Drake R. (2015). A review of Additive Manufacturing technology and Cost Estimation techniques for the defence sector. CIRP J. Manuf. Sci. Technol..

[7] Mehnen J., Ding J., Lockett H., Kazanas P. (2014). Design study for wire and arc additive manufacture. Int. J. Prod. Dev..

[8] Wang F., Williams S., Colegrove P., Antonysamy A.A. (2013). Microstructure and Mechanical Properties of Wire and Arc Additive Manufactured Ti-6Al-4V. Met. Mater. Trans. A Phys. Metall. Mater. Sci..

[9] Almeida P., Williams S. Innovative Process Model of Ti–6Al–4V Additive Layer Manufacturing Using Cold Metal Transfer (CMT). Proceedings of the 21st Annual International Solid Freeform Fabrication Symposium.

[10] Frazier W.E. (2014). Metal Additive Manufacturing: A Review. J. Mater. Eng. Perform..

[11] Ding D., Pan Z., Cuiuri D., Li H. (2015). Wire-feed additive manufacturing of metal components: Technologies, developments and future interests. Int. J. Adv. Manuf. Technol..

[12] Ding J., Martina F., Williams S. Production of Large Metallic Components by Additive Manufacture—Issues and Achievements. Proceedings of the 1st Metallic Materials and Processes: Industrial Challenges.

[13] Donoghue J.M., Antonysamy A.A., Martina F., Colegrove P., Williams S.W., Prangnell P. (2016). The effectiveness of combining rolling deformation with Wire–Arc Additive Manufacture on β-grain refinement and texture modification in Ti–6Al–4V. Mater. Charact..

[14] Martina F., Colegrove P.A., Williams S.W., Meyer J. (2015). Microstructure of Interpass Rolled Wire + Arc Additive Manufacturing Ti-6Al-4V Components. Met. Mater. Trans. A Phys. Metall. Mater. Sci..

[15] Ding D., Pan Z., Cuiuri D., Li H. (2015). A practical path planning methodology for wire and arc additive manufacturing of thin-walled structures. Robot. Comput. Integr. Manuf..

[16] Karlsson L. (2012). Welding duplex stainless steels—A review of current recommendations. Weld. World.

[17] Jebaraj A.V., Ajaykumar L., Deepak C., Aditya K. (2017). Weldability, machinability and surfacing of commercial duplex stainless steel AISI2205 for marine applications—A recent review. J. Adv. Res..

[18] Gunn R.N. Intermetallic Formation in Super Duplex Stainless Steel Heat Affected Zones. Proceedings of the 5th Duplex Stainless Steel World Conference.

[19] Olsson J., Snis M. (2007). Duplex—A new generation of stainless steels for desalination plants. Desalination.

[20] TMR Stainless (2014). Practical Guidelines for the Fabrication of Duplex Stainless Steels.

[21] Zhang Z., Jing H., Xu L., Han Y., Zhao L. (2016). Investigation on microstructure evolution and properties of duplex stainless steel joint multi-pass welded by using different methods. Mater. Des..

[22] Amigo V., Bonache V., Teruel L., Vicente A. (2006). Mechanical properties of duplex stainless steel laser joints. Weld. Int..

[23] Hsieh R.-I., Liou H.-Y., Pan Y.-T. (2001). Effects of Cooling Time and Alloying Elements on the Microstructure of the Gleeble-Simulated Heat-Affected Zone of 22% Cr Duplex Stainless Steels. J. Mater. Eng. Perform..

[24] Kang D.H., Lee H. (2012). Effect of Different Chromium Additions on the Microstructure and Mechanical Properties of Multipass Weld Joint of Duplex Stainless Steel. Metall. Mater. Trans. A.

[25] Rede V., Žmak I. Analysis of microstructure and microstructural changes in duplex steels. Proceedings of the 12th International Foundrymen Conference.

[26] Gu J., Ding J., Williams S.W., Gu H., Ma P., Zhai Y. (2016). The effect of inter-layer cold working and post-deposition heat treatment on porosity in additively manufactured aluminum alloys. J. Mater. Process. Technol..

[27] Stützer J., Totzauer T., Wittig B., Zinke -I.M., Jüttner S. (2019). GMAW Cold Wire Technology for Adjusting the Ferrite–Austenite Ratio of Wire and Arc Additive Manufactured Duplex Stainless Steel Components. Metals.

[28] Hosseini V.A., Högström M., Hurtig K., Valiente Bermejo M.A., Stridh L.-E., Karlsson L. (2019). Wire-arc additive manufacturing of a duplex stainless steel: Thermal cycle analysis and microstructure characterization. Weld. World.

[29] Eriksson M., Lervåg M., Sørensen C., Robertstad A., Brønstad B.M., Nyhus B., Aune R., Ren X., Akselsen O.M. (2018). Additive manufacture of superduplex stainless steel using WAAM. MATEC Web Conf..

[30] Adebayo A., Mehnen J., Tonnellier X. Limiting Travel Speed in Additive Layer Manufacturing. Proceedings of the 9th International Trends in Welding Research Conference.

[31] Xiong J., Yin Z., Zhang W. (2016). Closed-loop control of variable layer width for thin-walled parts in wire and arc additive manufacturing. J. Mater. Process. Technol..

[32] Xiong J., Lei Y., Chen H., Zhang G. (2017). Fabrication of inclined thin-walled parts in multi-layer single-pass GMAW-based additive manufacturing with flat position deposition. J. Mater. Process. Technol..

[33] Kazanas P., Deherkar P., Almeida P.L., Lockett H., Williams S. (2012). Fabrication of geometrical features using wire and arc additive manufacture. Proc. Inst. Mech. Eng. Part. B J. Eng. Manuf..

[34] Almeida P.M.S. (2012). Process Control and Development in Wire and Arc Additive Manufacturing. Ph.D. Thesis.

[35] Alvarez T.R., Pavarino M.R.C., De Souza G.C., Pardal J.M., Tavares S.S.M., Ferreira M.L.R., Filho I.C. (2016). Influence of interpass temperature on the properties of duplex stainless steel during welding by submerged arc welding process. Weld. Int..

[36] Posch G., Chladil K., Chladil H. (2017). Material properties of CMT—metal additive manufactured duplex stainless steel blade-like geometries. Weld. World.

[37] Hoefer K., Haelsig A., Mayr P. (2017). Arc-based additive manufacturing of steel components—Comparison of wire- and powder-based variants. Weld. World.

[38] Derekar K., Lawrence J., Melton G., Addison A., Zhang X., Xu L. (2019). Influence of Interpass Temperature on Wire Arc Additive Manufacturing (WAAM) of Aluminium Alloy Components. MATEC Web Conf..

[39] Pan Z., Pan Z., Ding D., Cuiuri D., Li H., Xu J., Norrish J. (2018). A review of the wire arc additive manufacturing of metals: Properties, defects and quality improvement. J. Manuf. Process..

[40] Cong B., Qi Z., Qi B., Sun H., Zhao G., Ding J. (2017). A Comparative Study of Additively Manufactured Thin Wall and Block Structure with Al-6.3%Cu Alloy Using Cold Metal Transfer Process. Appl. Sci..

[41] Ryan E., Sabin T., Watts J., Whiting M. (2018). The influence of build parameters and wire batch on porosity of wire and arc additive manufactured aluminium alloy 2319. J. Mater. Process. Technol..

[42] Melfi T. (2010). New code requirements for calculating heat input. Weld. J..

[43] Do Nascimento A.S., Batista M.A., do Nascimento V.C., Scotti A. (2007). Assessment of electrical power calculation methods in arc welding and the consequences on the joint geometric, thermal and metallurgical predictions. Soldag. Insp..

[44] Joseph A., Harwig D., Farson D.F., Richardson R. (2003). Measurement and calculation of arc power and heat transfer efficiency in pulsed gas metal arc welding. Sci. Technol. Weld. Join..

[45] Cho M.H., Farson D.F. (2007). Understanding bead hump formation in gas metal arc welding using a numerical simulation. Met. Mater. Trans. A.

[46] ISO 25178-2:2012 (2012). Geometrical Product Specifications (GPS)—Surface Texture: Areal—Part 2: Terms, Definitions and Surface Texture Parameters.

[47] Aver’Yanova I.O., Bogomolov D.Y., Poroshin V.V. (2017). ISO 25178 standard for three-dimensional parametric assessment of surface texture. Russ. Eng. Res..

[48] Hosseini V.A., Hurtig K., Eyzop D., Östberg A., Janiak P., Karlsson L. (2019). Ferrite content measurement in super duplex stainless steel welds. Weld. World.

[49] Putz A., Althuber M., Zelić A., Westin E.M., Willidal T., Enzinger N. (2019). Methods for the measurement of ferrite content in multipass duplex stainless steel welds. Weld. World.

[50] Ding J., Colegrove P., Martina F., Williams S.M., Wiktorowicz R., Palt M. (2015). Development of a laminar flow local shielding device for wire + arc additive manufacture. J. Mater. Process. Technol..

[51] Ma Y., Cuiuri D., Li H., Pan Z., Shen C. (2016). The effect of postproduction heat treatment on γ-TiAl alloys produced by the GTAW-based additive manufacturing process. Mater. Sci. Eng. A.

[52] Sedlaček M., Podgornik B., Vižintin J. (2009). Influence of surface preparation on roughness parameters, friction and wear. Wear.

[53] Lee B.H., Lee H.W., Shin Y.T. (2015). Intergranular Corrosion Characteristics of Super Duplex Stainless Steel at Various Interpass Temperatures. Int. J. Electrochem. Sci..

[54] ISO 6520-1:2007 (2007). Welding and Allied Processes—Classification of Geometric Imperfections in Metallic Materials—Part 1: Fusion Welding.

[55] ISO 5817:2014 (2014). Welding—Fusion-Welded Joints in STEEL, Nickel, Titanium and Their Alloys (Beam Welding Excluded)—Quality Levels for Imperfections.

[56] Jiang Z., Li H.-B., Chen Z., Huang Z., Zou D., Liang L. (2016). The Nitrogen Solubility in Molten Stainless Steel. Steel Res. Int..

[57] Cherry J.A., Davies H.M., Mehmood S., Lavery N., Brown S.G.R., Sienz J. (2014). Investigation into the effect of process parameters on microstructural and physical properties of 316L stainless steel parts by selective laser melting. Int. J. Adv. Manuf. Technol..

[58] Stiles D.J. (2005). Effect of porosity on thermal response, hardness, hardenability and microstructure of powder metallurgy steels. Surf. Eng..

[59] Tucho W.M., Lysne V.H., Austbø H., Sjolyst-Kverneland A., Hansen V. (2018). Investigation of effects of process parameters on microstructure and hardness of SLM manufactured SS316L. J. Alloy. Compd..

[60] EN 10088-3:2014 (2014). Stainless Steels—Part 3: Technical Delivery Conditions for Semi-Finished Products, Bars, Rods, Wire, Sections and Bright Products of Corrosion Resisting Steels for General Purposes.

[61] EN 10088-5:2009 (2009). Stainless Steels—Part 5: Technical Delivery Conditions for Bars, Rods, Wire, Sections and Bright Products of Corrosion Resisting Steels for Construction Purposes.

